# A Computational Model of the Brain Cortex and Its Synchronization

**DOI:** 10.1155/2020/3874626

**Published:** 2020-10-23

**Authors:** Sadeem Nabeel Saleem Kbah

**Affiliations:** ^1^Biomedical Engineering Department, Al-Khwarizmi College of Engineering, University of Baghdad, Baghdad, Iraq 10071; ^2^Istanbul Technical University, Istanbul, Turkey

## Abstract

Obtaining the computational models for the functioning of the brain gives us a chance to understand the brain functionality thoroughly. This would help the development of better treatments for neurological illnesses and disorders. We created a cortical model using Python language using the Brian simulator. The Brian simulator is specialized in simulating the neuronal connections and synaptic interconnections. The dynamic connection model has multiple parameters in order to ensure an accurate simulation (Bowman, 2016). We concentrated on the connection weights and studied their effect on the interactivity and connectivity of the cortical neurons in the same cortical layer and across multiple layers. As synchronization helps us to measure the degree of correlation between two or more neuronal groups, the synchronization between the neuronal groups, which are connected across layers, is considered. Despite its obvious importance, there are no sufficient studies concerned about the synchronization in the simulated cortical models. Such studies can help in examining the hypothesis and the dynamical behavior of the simulated model. In this paper, we simulated a cortical model and dynamical behavior and then studied the effect of input noise on its internal neuronal networks and their synchronization.

## 1. Introduction

In the last decade, the work on obtaining computational models for the brain has been increased tremendously. To understand the connectivity and functionality of the neuronal groups, models at different levels have been proposed. Especially, modeling the cortex has been the most studied subject among the computational neuroscientist. Mostly, the focus is on improving the measurement and imaging techniques like EEG (electroencephalogram) and fMRI (functional magnetic resonance imaging) and collecting and processing data with these techniques. These modern brain imaging techniques are valuable as they provide data for the approval of the computational models focusing on understanding the relationship between cognition and the brain [1–3]. The models developed for the cortex are ranging from a detailed single neuron model [4] to the mass models where the collective activity of a population of the neuron is considered [3].

Still, the field of brain modeling is in development and needs more ideas to improve the already available models and theories. The most accurate modeling of the human brain occurred in a Japanese university; using a Japanese supercomputer, it took 40 minutes to simulate 1 percent of the human brain neuronal connectivity working for 1 second [2]. The modeling of the brain, at different levels, would provide means to understand the cognitive processes and improve new approaches for diagnosis and treatment of neurological diseases and disorders. Of course, as nature inspires engineers, work on the brain would provide new ideas to develop intelligent agents and systems. New approaches in machine learning already are due to work on the brain as Barto et al.'s reinforcement learning [5, 6] and Grossberg's adaptive resonance theory [7]. The spiking neuron models are also considered in developing new machine learning methods [8], and working on the spiking neural network models would improve these approaches.

In this work, the activities of a cortical population will be considered, and the model will be constructed using the Izhikevich neuron model; it has been recorded in all mammal brains in a band starting from as low as one wave every forty seconds (0.025 Hz) to as high as six hundred waves per second (600 Hz) [9]. Since the synchronization of neuronal activity is considered to give rise to brain oscillations observed in EEG [10, 11], synchronization of the neuronal groups will be investigated. Synchronization is the degree of correlation between two or more spiking neuron activities in the same time interval and it depends on many chemical and electrical spiking interactions [12].

The measurement of synchronization has many applications in monitoring the activity of the brain and the degree of interaction between different brain parts, which gives us a thorough idea about brain functionality both normal and deficiency, where synchronization can give us a right indication about the degree of the engagement of two or more neuronal groups in some mental task, and the more coherence between the firing patterns of these neuronal groups, and the physiological interactivity between them [4].

In this way, we can state that the degree of synchronization can be an indication of how good the mental activities of a person are and also can be used as an indication of the spreading of some mental diseases such as epilepsy, Parkinson's disease, autism, and Alzheimer's disease (AD) [13–15].

From the engineering point of view, the synchronization index is an important indication of the dynamical behavior of nonlinear large network systems, since in the case of computational modeling, we are more interested in the overall behavior of the entire system. On the other hand, as we know the brain consists of anatomically separated parts called lobes. Physiologically, it is found that these lobes can be engaged together to do a certain mental task [16].

Although of the obvious importance of synchronization, there are no studies about the synchronization of the simulated neuronal models. In this paper, we simulated the brain cortex and its dynamical behavior and calculated the occurred synchronization between the internal neuronal groups due to implying thalamic noise as a stimulus. Calculating the synchronization between the internal neuronal groups of our model will not just ensure the stability of the model [17] but will also ensure the right interconnectivity and dynamical behavior of the simulated model. This latter property can be helpful when using this model to study cortical behavior through different types of learning and other applications.

In this work, we are inspired by Izhikevich's and Edelman's work on a large-scale brain model [18], and we simulated 10000 neuron activities where 22 different types of multicompartmental neurons are considered. The model is constructed with the Brian (v1.4) simulator, and this work gives an example of realizing a large-scale model of the brain using a computer with ordinary computational properties [5]. The results obtained with the model are similar to neuronal activity observed with EEG measurements of the normal human brain cortex. Using this model, we investigated the effect of changing the connection weights on synchronization. It is claimed that the relation between synchrony and the neuronal code aroused from the activation of a group of neurons will inspire new learning techniques [7]. This model can be used also to study the role of short-term plasticity and STDP (spike timing-dependent plasticity) on learning as in [5, 19], where the Brian simulator has a library that is related to STDP, by defining the pre- and postsynaptic neuronal groups, which can be applied to our model [20].

## 2. Firing Patterns in the Cortex and Simple Neuron Model

The cortex has an important role in the functionality of the brain. It includes the sensory areas, motor areas, and the association areas, and has interactions with the subcortical areas. All these make the cortex responsible for most of the activities of our daily life. Association areas are especially important, in combining different sensory inputs with the processing of subcortical areas to give rise to high-order cognitive tasks. The association functions include generally everything related to language, analyzing information, memorizing, planning, and conscious thinking (creativity) [21].

The cortex consists of six layers; each one of these layers has different cell types with its own firing type which are listed in Table 1; we worked to simulate the brain cortex both anatomically (by using the same rate of the existence of neurons of the cortex and simulated number of synapsis between them) and physiologically (by simulating their group behavior) [22, 23]. On the other hand, spiking in the cortical layers can be divided functionally into excitatory and inhibitory groups. While the excitatory groups work to increase the sympathetic activity in the body, the inhibitory groups work to increase the parasympathetic actions in the body [24].

To model the different behaviors of different cell types in the cortex, we used Izhikevich's neuron model, which is expressed mathematically through the use of the following nonlinear dynamical equations [23].

(1) $$\begin{array}{c} \frac{dv}{dt}=0.04v^{2}+5v+140-u+I, \end{array}$$

(2) $$\begin{array}{c} \frac{du}{dt}=a\left(bv-u\right). \end{array}$$

There is a reset condition:

(3) $$\begin{array}{c} \begin{array}{c} \text{If }v>30\text{ mV},\text{then},\\ v=c,\\ u=u+d, \end{array} \end{array}$$

where *v* and *u* are dimensionless variables, with *v* representing the membrane potential and *u* representing the membrane recovery variable.

The Izhikevich model is capable of expressing different firing types due to chance in the parameter values; so for a large-scale model of the cortex, it provides a suitable model of neuron behavior. Equations (2) and (3) include also other parameters (*a*, *b*, *c*, and *d*); these parameters are responsible on giving the spike its characteristic shape and can be found in Table 2 for each spiking type.

## 3. Creating the Cortical Layers

As we mentioned previously, the cortical brain neurons can be classified functionally to excitatory and inhibitory neurons. The distribution of these two different functional groups of cells is 80% excitatory and 20% inhibitory [25]. In the cortex model which we proposed, the excitatory cells are pyramidal and spiny cells, and their distribution with the spiking type is illustrated in Table 3, according to the level considered. The cortex also has two types of inhibitory cells which are called GABA cells due to the effective neurotransmitter. Like excitatory neurons, they are also distributed in all six layers [16]; the way they are considered in the proposed model is illustrated in Table 3. From Tables 3 and 4, we can see that the percentage of excitatory and inhibitory neurons considered in the model matches the human cortex [25].

Up to this point, we have discussed the neuron types that are considered in the proposed model where pyramidal and spiny cells are taken as excitatory neurons, and GABAergic _(nb, b)_ neurons represent the inhibitory. Now, to simulate this cortical model, these neurons in the different layers should be connected according to connection weights and their sparseness. In Brian environment, the connections are defined with certain *weight* and *sparseness* values as dynamic variables and this connection is defined as follows [26]:

Connection (RS_p2, LTS_nb2,' g_ampa',

structure =' sparse', sparseness = 0.1,

weight = 10)

where RS_p2_ denotes the group from which spikes will be propagated, LTS_nb2_ denotes the group to which spikes will be propagated.

With this structure, it is possible to define the types of connections between the connected layers to be either *sparse* or *dense*. *Sparse* which means a spike will either occur or not between the connected layers compared to the *dense* which means a spike will certainly occur between all the connected layers; it will be the default choice in our case since it is more suitable for modifying the dynamical connections [27, 28]. The dynamical part of the synaptic connections (here for the groups, in our example it is *g*_ampa_) is represented by another differentiation equation, Equation (5), where *g*_ampa_ denotes the dynamic variable corresponding to synaptic connection and it is initialized with random values. *τ*_ampa_ is the recovery time, which is the time required by the membrane to return to its rest potential; each neuronal group has its own dynamical variable and recovery time (*τ*). The whole types of dynamic variables (*g*) used in our model are listed in Table 5.

(5) $$\begin{array}{c} =-. \end{array}$$

Returning to the example of connection we mentioned, *sparseness* denotes the probability of a connection between two groups of neurons to occur and its value is taken to be between 0.1 and 0.2 in our model [25]. The synaptic *weight* between the neurons can also be changed dynamically, where the *weight* here denotes the values added to the dynamical variable (*g*) of each connection in order to increase or decrease the strength of the connection as in Equation (6).

(6) $$\begin{array}{c} \Delta g=g+\Delta W. \end{array}$$

Once connected, neurons start to fire; the strength of the synaptic connections between them will either increase or decrease according to the functional property of the neurons considered (excitatory neurons or inhibitory neurons). While excitatory neurons increase the synaptic connections, the inhibitory neurons decrease them. These changes in the synaptic connections due to firing of neurons are called Hebbian rule and it is specified by changing the weights of connections. Where according to the definition of Hebbian learning and according to general Equation (7), STDP is a good choice for long-term synaptic plasticity exploiting, where spike timing-dependent plasticity (STDP) is a Hebbian synaptic learning rule in which the millisecond timing of pre- and postsynaptic-paired activities induces either synaptic long-term potentiation (LTP) or long-term depression (LTD) [29].

(7) $$\begin{array}{c} \Delta w_{i}=\eta x_{i}y, \end{array}$$

where *w*_*i*_ is the change in synaptic weight, *η* is the learning rate, *x*_*i*_ is the presynaptic neuron, and *y* is the postsynaptic neural response.

Also about the weight values, it has been taken from 0 to 100 for statistical purposes. In our model, the *weight* will be changed throughout iterations to investigate its role in synchronization. We took the *weight* values to be between 1 and 100. Now, as we defined our neuron structures and connections, we are ready to form the neuronal groups that compromise the activity of the cortex.

## 4. Creating Neuronal Groups

In order to model the behavior of the cortex and corticothalamic connections, the most effective firing pattern for each layer is considered and parameters of the Izhikevich neuron model are chosen to create the relative firing pattern. The neurons in each group are not only connected to the neurons in the group but also to the neurons in other groups. Even though the connection between and in groups has different properties, still the establishment of connections is done in such a manner that with the parameters defining connections in Brian, the biologically plausible model is obtained.

The thalamic model was also built, where it consists of both the excitatory and inhibitory groups. While the excitatory groups consist of regular spiking (RS) and bursting (IB), the inhibitory groups consist of the GABA groups (see Tables 3 and 4). The connections of the thalamic groups were targeted towards cortical layers of L2/3, L4, and L6. The effect of the corticothalamic connections was introduced as a noise input to the cortical model at the mentioned layers.

**Figure 1:**
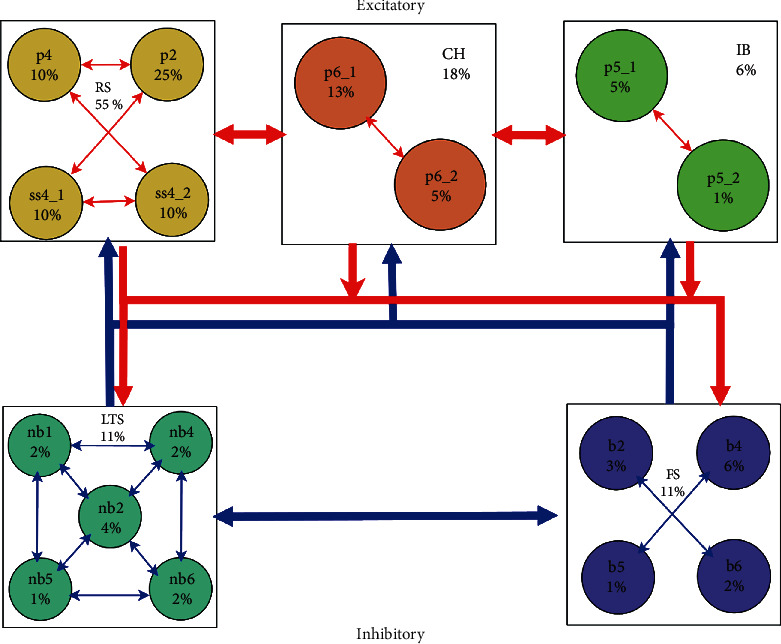
The entire connections between the cortical neuronal groups, where the three upper blocks (RS, CH, IB) are the excitatory groups, while the lower two (FS, LTS) are the inhibitory ones. The sum of total percentage is 80% for the excitatory groups and 20% for the inhibitory groups, respectively (more details about the constituents of each group are shown in Tables 3 and 4). Arrows are colored as *red* for excitatory and *blue* for inhibitory. As we can see, the connections are established in such a way that every single neuron can connect to its followers in the same group and the neurons in other groups via bidirectional connections.

The entire network proposed for the cortex with connections properties and different cortical neuronal groups is shown in Figure 1. Here, each one of the five blocks represents a neuronal group; inside each block is the name of the group and its connection sparseness is given as a percentage. One can notice that the names of the blocks are the same as the names of spiking types mentioned in Table 1. The circles inside each block stand for the subgroups of the main group, which represents the percentage of distribution of the neurons. The main group in each layer in the cortex can be realized from the name of each circle which coincides with Tables 3 and 4; each subgroup has its own connection properties defined with the parameters of dynamic variable *g* as illustrated in Table 4.

The three upper blocks (RS, CH, IB) are the excitatory groups, while the lower two (FS, LTS) are the inhibitory ones, with the sum of total percentage of 80% for the excitatory groups and 20% for the inhibitory groups, respectively, as shown in Tables 3 and 4. Arrows represent connections, and as we can see, the connections are established in such a way that every single neuron has the ability to connect to its own followers in the same group and the neurons in other groups via bidirectional connections. The arrows are colored as red for excitatory and blue for inhibitory. In this way, we can make a model that is capable of simulating the behavior of microcolumns in the cortex, even in its learning capabilities [30].

**Figure 2:**
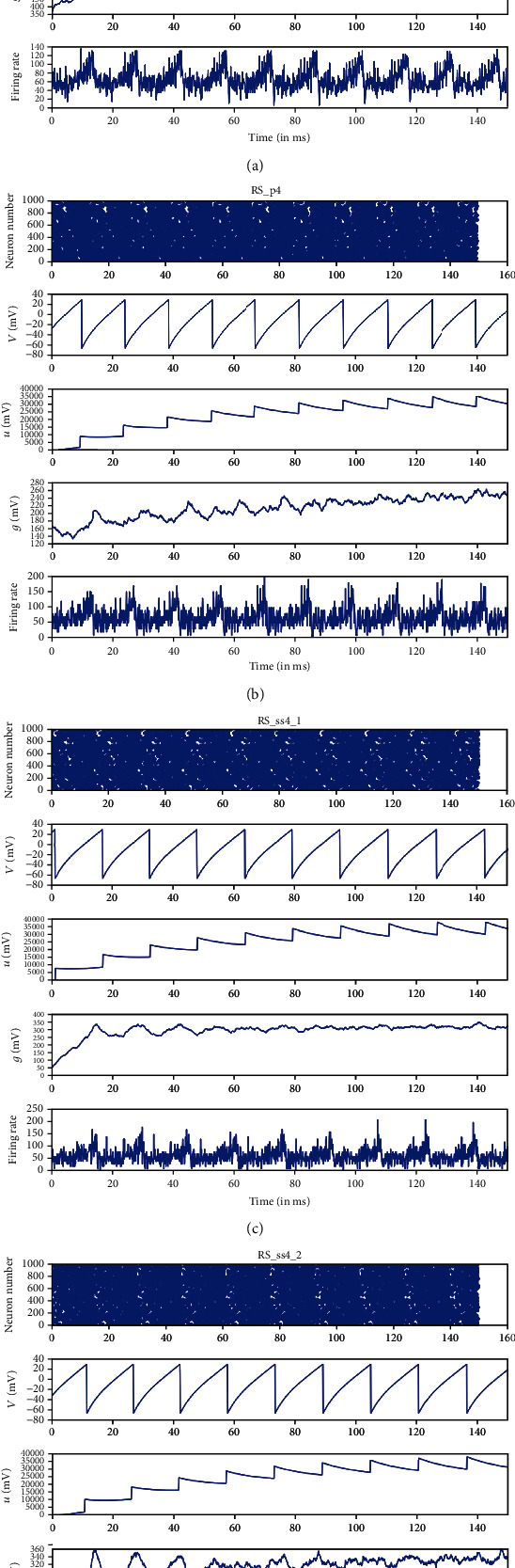
Some of the simulation results of the excitatory groups. (a) RS_p2_ illustrates the spiking type which is also the name of a neuronal group in our model, while the subscript (p2) means pyramidal cells located in layer (2) in the cortex (for more information about the excitatory groups in this model, please refer to Table 3). The first row represents the raster plot of the firing neurons and their temporal location per time (in our model 1 ms), with the *y*-axis (0-2500) represents the number of neurons. Here, each dot represents a firing neuron, and by comparing the dot against *y*-axis, we can know its number in the group (0-2500), and the time of firing can be read from the *x*-axis (0-150); ms is the time of simulation process. The second row is the plot of membrane voltage of a single neuron which in our case is (RS) type (different spiking groups had been created according to the parameters shown in Table 2). The third row is variable *u*, the recovery time or refractory period of this neuronal group, which is the time required by the neuron membrane to return to its reset potential after each spike; the fourth row is the dynamic variable *g*, and finally, the fifth row is the firing rate which represents the number of spiking neurons per unit time which in our case milliseconds (ms). This stands for all the other plots (b, c, and d) in this figure which are the simulation output of the groups (RS_P4_, RS_ss4_1__, and RS_ss4_2__).

**Figure 3:**
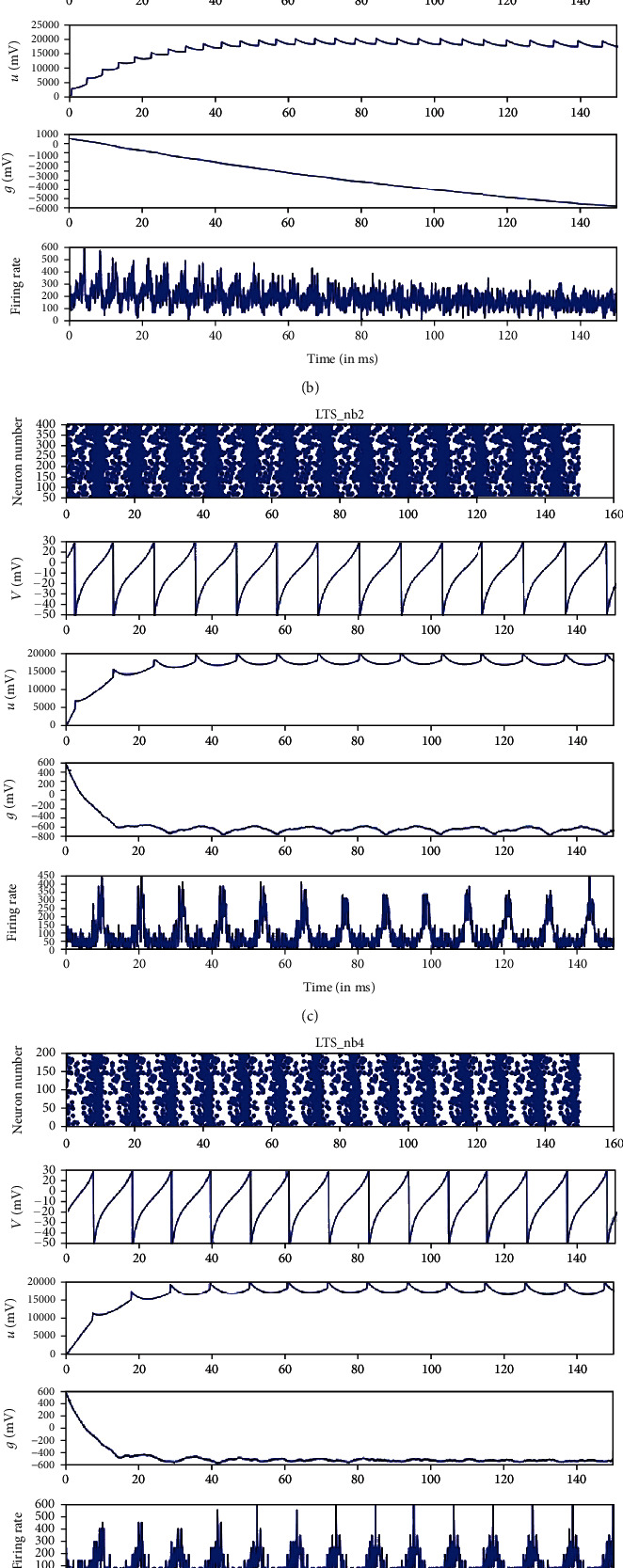
Some of the simulation results of the inhibitory groups. The involved groups are FS_b2_, FS_b4_, LTS_nb2_, LTS_nb4_, and LTS_nb5_; more details about the inhibitory groups used in this model can be found in Table 4. (a) FS_b2_ illustrates the spiking type which is also the name of a neuronal group in our model, while the subscript b means GABA type b cells located in layer (2) in the cortex. The first row represents the raster plot of the firing neurons and their temporal location per time (in our model 1 ms), with the *y*-axis (0-2500) represents the number of neurons. Here, each dot represents a firing neuron, and by comparing the dot against *y*-axis, we can know its number in the group (0-2500), and the time of firing can be read from the *x*-axis (0-150); ms is the time of simulation process. The second row is the plot of membrane voltage of a single neuron which in our case is (FS) type (different spiking groups had been created according to the parameters shown in Table 2). The third row is variable *u*, the recovery time or refractory period of this neuronal group, which is the time required by the neuron membrane to return to its reset potential after each spike; the fourth row is the dynamic variable *g*, and finally, the fifth row is the firing rate which represents the number of spiking neurons per unit time which in our case milliseconds (ms). This stands for all the other plots (b, c, d, and e) in this figure.

The results obtained from the simulation of the model are given in Figures 2 and 3, where the title of each figure (RS_p2_ in Figure 2 for example) illustrates the spiking type which is also the name of the neuronal group, while (p2) means pyramidal cells located in layer (2) in the cortex (see Tables 3 and 4). In Figures 2 and 3, some of the excitatory and inhibitory groups are plotted, where the first row is the raster plot of the firing neurons which shows us the overall activity of the groups by showing the number and temporal location of the firing neuron in each unit time (in our model 1 ms), the second row is the single spike (the membrane voltage of one neuron) which in our case is (RS) type in Figure 2, the third row is variable *u* of this neuronal group which is the time recovery after each spike, the fourth row is the dynamic variable *g*, and finally, the fifth row is the firing rate which represents the number of spiking neurons per unit time which in our case milliseconds (ms). So, from Figures 2 and 3, we can get the overall activity of the groups which is given by raster plots; the behavior of single neuron, the dynamics of the connections, and the firing rate illustrate activeness in the neuronal population which are also shown.

If we take a second look at Figures 2 and 3 and by examining the first and fifth rows, we can see that the spiking of the group has a random behavior compared to the single spiking behavior shown in the second row of the same figures. This behavior is a sign of existence of an interaction between different groups in the model, i.e., this kind of randomness in rows (1 and 5) means that the spiking of this group of neurons has been affected by the spikes of other groups since normally these two rows were to show more rhythmic spiking behavior that match with the single spike behavior shown in the second rows of the figures if there was no synchrony at all. This interaction between this group and the other groups can be measured then by measuring the degree of synchrony that occurred between them. In the next section, we will calculate how much each group of neurons has been affected by other groups and by the weight of the connections and this will be done by calculating the synchrony measuring factor. The obtained results represent both the cortical model, the thalamic model, and their connection dynamical behavior.

## 5. Measuring Synchrony

Finally, in order to ensure the biological postulate of our model, one way is to monitor its dynamical behavior through the synchronization measurement. To decide on the level of synchronization, a synchronization measurement has to be considered. Especially, to see the synchronization between groups, the interactivity of each one of the neuronal groups created with other groups is considered while changing the connection weights between the groups. This will help us to understand the effect of connection weights on the whole model, the degree of effectiveness of each layer by the action of the other layers. The synchronization measurement can be considered as the estimator of connectivity in the cortex [18]. Measuring the interactivity between cortical layers is important especially in the case of cognitive acts, which need a harmonic and cooperative action from most of the cortical layers [31]; it is important also in many applications like detecting and measuring the degree of brain disorders [32]. The synchrony measurement of the neuronal groups increases the ability to predict the behavior for long-term neuronal interaction. It can also give us a prediagnostic about the availability of specific mental diseases [33]. Furthermore, it helps the investigation of the active brain areas during some activities like movement or cognition [26, 34]. The synchrony measure for neuronal activity of *N* neurons is given as follows [34]:

(8) $$\begin{array}{c} X^{2}\left(N\right)=\frac{\sigma_{V}^{2}}{\left(1/N\right)\sum_{i=1}^{N}‍\sigma_{V_{i}}^{2}}. \end{array}$$

In Equation (8), *σ*_*V*_^2^ is the variance of the activity of all neurons; so to calculate it, the mean of *N* neuron behavior is considered and the signal *V*(*t*) is defined as follows:

(9) $$\begin{array}{c} V\left(t\right)=\frac{1}{N}\overset{N}{\underset{i=1}{\sum }}‍v_{i}. \end{array}$$

So, the behavior of *V*(*t*) averaged over *N* is calculated first; then, *σ*_*V*_ is obtained as follows:

(10) $$\begin{array}{c} \sigma_{V}^{2}={\langle {\left[V\left(t\right)\right]}^{2}\rangle }_{t}-{\left[{\langle V\left(t\right)\rangle }_{t}\right]}^{2}. \end{array}$$

The variance of each neuron is calculated in a similar way:

(11) $$\begin{array}{c} \sigma_{V_{i}}^{2}={\langle {\left[V_{i}\left(t\right)\right]}^{2}\rangle }_{t}-{\left[{\langle V_{i}\left(t\right)\rangle }_{t}\right]}^{2}. \end{array}$$

**Figure 4:**
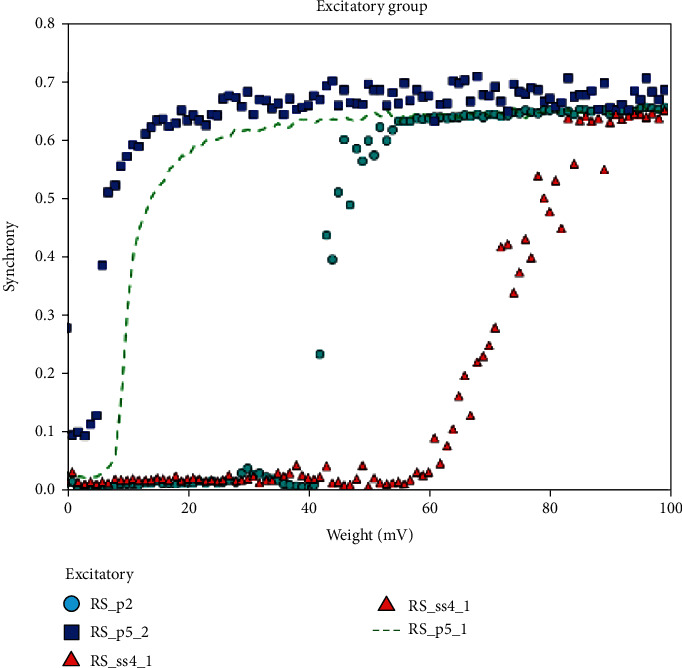
Some of the simulation results of *synchronization measurements* of the excitatory groups. The included groups are (RS_p2_, RS_p5_2__, RS_p5_2__, and RS_ss4_1__). Synchronization index normally is between 0 and 1, where 0 means no synchronization at all, while 1 shows a full synchronization. The obtained figure tends towards 1 as the weight on connection increases. So, the figure shows an increasing in the synchronization between the excitatory groups as the weight of connections (or the interaction between groups) increases. We notice that RS_p5_2__ has reached the synchronization before the others, while the last one, as the weight of the connections increases, was RS_ss4_1__.

**Figure 5:**
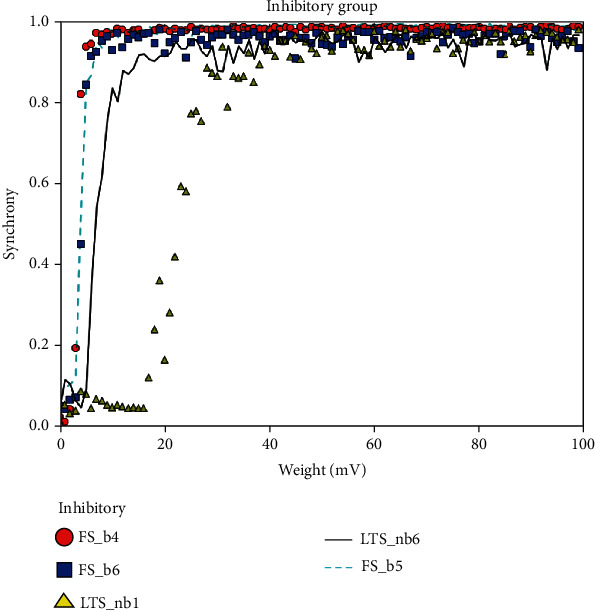
Some of the simulation results of *synchronization measurements* of the inhibitory groups.

The synchrony measure *X* is calculated while changing the connection weights in an interval of 1 to 100 incrementing each time by 10 mV for the inhibitory and excitatory groups. Regular spiking (RS) and chattering (CH) have neuron behavior in the excitatory group, while the inhibitory group has fast spiking (FS) neurons. The simulation results are given in Figures 4 and 5.

Figures 4 and 5 show the results of synchronization measurement of each group. In Figure 4, we plotted the synchrony measurements of the excitatory groups (RS_p2_, RS_p5_2__, RS_ss4_1__, and RS_p5_1__), while in Figure 5, we plotted the synchrony measurements of the inhibitory groups (FS_b4_, FS_b6_, LTS_nb1_, LTS_nb6_, and FS_b5_). The *y*-axis is the degree of synchronization which is normally between 0 and 1, where 0 means no synchrony at all and 1 means total synchronization between this group (under study) and the other groups in the model. The *x*-axis is the value of weight of connection; it changes during the run in each iteration.

Generally, the figures show good synchronization between the neuronal groups in our model, which means there is good connectivity between them.

## 6. Conclusion

In this simulated model, we worked to simulate the brain cortex both anatomically (by using the same rate of the existence of neurons of the cortex and simulated number of synapsis between them) and physiologically (by simulating their group behavior). The effect of connection weights over the strength of the connection is investigated. The degree of connectivity was tested thoroughly by calculating the synchronization between the connected neuronal groups. The synchronization can give us a right indication and a thorough idea about brain functionality both in normal and deficiency cases, and about the degree of the engagement of two or more neuronal groups in some mental task, it also serves as an index to indication of the dynamical behavior of nonlinear large network systems, since in the case of computational modeling, we are more interested in the overall behavior of the entire system, which here can justify the simulated model and ensures its, biological-like, dynamical behavior. Even though synchronization within a group has been considered in previous works, the synchronization between groups is considered for the first time in this work up to our knowledge. The result shows that there is an increase in the synchronization factor with the increase occurs in the connection weights, which shows that there is an increase in the connectivity between neurons. This conclusion can be used in multiple applications especially in machine learning and cognitive research fields. As a future work, this model can be improved and used in the field of AI learning, especially the STDP method.

## Figures and Tables

**Table 1:** Type of spikes available in the brain cortex.

Spiking type symbol	Spiking type name
RS	Regular spiking
IB	Intrinsically bursting
CH	Chattering
FS	Fast spiking
LST	Low-threshold spiking

**Table 2:** List of parameters *a*, *b*, *c*, and *d* used in Equations (2) and (3).

Spiking type symbol	Values of parameters			
	*a*	*b*	*c*	*d*
RS	0.02/ms	0.2/ms	−65∗mV	8∗volt/second
IB	0.02/ms	0.2/ms	−55∗mV	4∗volt/second
CH	0.02/ms	0.2/ms	−50∗mV	2∗volt/second
FS	0.05/ms	0.2/ms	−50∗mV	2∗volt/second
LST	0.1/ms	0.25/ms	−50∗mV	2∗volt/second

**Table 3:** Excitatory groups and its neuron information.

Type of neurons	Existence in the cortex	Actual number of neurons used in our model	Percentage of existence	Spiking type
Pyramidal cells	L2/3	2500	25%	RS
Pyramidal cells	L4	1000	10%	RS
Pyramidal cells	L2/3	500	5%	CH
Pyramidal cells	L5/6	100	1%	IB
Pyramidal cells	L4	1400	14%	CH
Pyramidal cells	L5/6	500	5%	IB
Spiny cells	L4	1000	10%	RS
Spiny cells	L2/3	1000	10%	RS
Total number of all excitatory neurons		8000	80%	

**Table 4:** Inhibitory groups and its neuron information.

Type of neuron	Existence in the cortex	Actual number of neurons used in our model	Percentage of existence	Spiking type
GABA_nb_	L1	200	2%	LTS
GABA_nb_	L2	400	4%	LTS
GABA_nb_	L4	200	2%	LTS
GABA_nb_	L5	100	1%	LTS
GABA_nb_	L6	200	2%	LTS
GABA_b_	L2	200	2%	FS
GABA_b_	L4	400	4%	FS
GABA_b_	L5	100	1%	FS
GABA_b_	L6	200	2%	FS
Total number of all inhibitory neurons		2000	20%	

**Table 5:** The dynamic variable *g* used in this model.

Neuron cell	Dynamic variable *g*	Type of action
Pyramidal neurons (L2, L4)	AMPA	Excitatory
Pyramidal neurons (L3, L5, L6)	NMDA	Excitatory
Spiny neurons	AMPA	Excitatory
GABA_nb_, GABA_b_	GABA	Inhibitory

## Data Availability

No data were used to support this study.

## References

[1] Bowman G. R. (2016). Accurately modeling nanosecond protein dynamics requires at least microseconds of simulation. *Journal of Computational Chemistry*.

[2] Urgen B. A., Pehlivan S., Saygin A. P. (2019). Distinct representations in occipito-temporal, parietal, and premotor cortex during action perception revealed by fmri and computational modeling. *Neuropsychologia*.

[3] Jaffe-Dax S., Boldin A. M., Daw N. D., Emberson L. L. (2020). A computational role for top–down modulation from frontal cortex in infancy. *Journal of Cognitive Neuroscience*.

[4] Maisel B., Lindenberg K. (2020). Channel noise effects on neural synchronization. *Physica A: Statistical Mechanics and its Applications*.

[5] Dafflon J., Pinaya W. H. L., Turkheimer F. (2019). Analysis of an automated machine learning approach in brain predictive modelling: a data-driven approach to predict brain age from cortical anatomical measures. *arXiv preprint arXiv*.

[6] Alexandre F. (2019). A computational model to study the dynamics of representations of rewards in the orbital and medial frontal cortex.

[7] Tripathi A., Geetika S., Singh K. K., Maurya P. K. Review of unsupervised adaptive resonance theory.

[8] Paugam-Moisy H., Bohte S. (2012). Computing with spiking neuron networks. *Handbook of Natural Computing*.

[9] Watson B. O., Buzsáki G. (2015). Sleep, memory & brain rhythms. *Daedalus*.

[10] Zhang H., Yang J., Xiang T. (2019). State-dependent spike and local field synchronization between the thalamic parafascicular nucleus and the dorsal striatum in a rat model of Parkinson’s disease. *Neuroscience*.

[11] Wang P., Goschl F., Friese U., Konig P., Engel A. K. (2015). Large-scale cortical synchronization promotes multisensory processing: an eeg study of visual-tactile pattern matching. *bioRxiv*.

[12] Deco G., Jirsa V. K., Robinson P. A., Breakspear M., Friston K. (2008). The dynamic brain: from spiking neurons to neural masses and cortical fields. *PLoS Computational Biology*.

[13] Palva S., Palva J. M. (2016). The role of local and large-scale neuronal synchronization in human cognition. *In Multimodal Oscillation-Based Connectivity Theory*.

[14] Boaretto B. R. R., Budzinski R. C., Prado T. L., Kurths J., Lopes S. R. (2018). Suppression of anomalous synchronization and nonstationary behavior of neural network under small-world topology. *Physica A: Statistical Mechanics and its Applications*.

[15] Babiloni C., Lizio R., Marzano N. (2016). Brain neural synchronization and functional coupling in Alzheimer’s disease as revealed by resting state eeg rhythms. *International Journal of Psychophysiology*.

[16] Budzinski R. C., Boaretto B. R. R., Prado T. L., Lopes S. R. (2019). Synchronization domains in two coupled neural networks. *Communications in Nonlinear Science and Numerical Simulation*.

[17] Budzinski R. C., Boaretto B. R. R., Prado T. L., Lopes S. R. (2017). Detection of nonstationary transition to synchronized states of a neural network using recurrence analyses. *Physical Review E*.

[18] Izhikevich E. M., Edelman G. M. (2008). Large-scale model of mammalian thalamocortical systems. *Proceedings of the National Academy of Sciences*.

[19] Ercelik E., Sengor N. S. A neurocomputational model implemented on humanoid robot for learning action selection.

[20] Spike-timing-dependent plasticity, howpublished. https://brian.readthedocs.io/en/1.4.2/stdp.html.

[21] Tripathi P., Sieber F. (2020). The adult central nervous system: anatomy and physiology. *Essentials of Neurosurgical Anesthesia & Critical Care: Strategies for Prevention, Early Detection, and Successful Management of Perioperative Complications*.

[22] García-Cabezas M. Á., Zikopoulos B., Barbas H. (2019). The structural model: a theory linking connections, plasticity, pathology, development and evolution of the cerebral cortex. *Brain Structure and Function*.

[23] Izhikevich E. M. (2003). Simple model of spiking neurons. *IEEE Transactions on Neural Networks*.

[24] Crisler R., Johnston N. A., Sivula C., Budelsky C. L. (2020). Functional anatomy and physiology. *The Laboratory Rat*.

[25] Wang X.-J. (2010). Neurophysiological and computational principles of cortical rhythms in cognition. *Physiological Reviews*.

[26] Goodman D., Brette R. (2008). Brian: a simulator for spiking neural networks in python. *BMC Neuroscience*.

[27] Kbah S. N. S. Investigation of a moderate cortical model synchronization created using brian simulator.

[28] Kbah S. N., Abushaeer M. T. Studing the emotional behaviour using a orbitocortical-amygdalo computational model.

[29] Sgritta M., Locatelli F., Soda T., Prestori F., D'Angelo E. U. (2017). Hebbian spike-timing dependent plasticity at the cerebellar input stage. *The Journal of Neuroscience*.

[30] Hawkins J., Ahmad S., Cui Y. (2017). A theory of how columns in the neocortex enable learning the structure of the world. *Frontiers in neural circuits*.

[31] Uzuntarla M., Torres J. J., Calim A., Barreto E. (2019). Synchronization-induced spike termination in networks of bistable neurons. *Neural Networks*.

[32] Uhlhaas P. J., Singer W. (2006). Neural synchrony in brain disorders: relevance for cognitive dysfunctions and pathophysiology. *Neuron*.

[33] Kbah S. N. S., Sengor N. S. Investigating the synchronization of cortical neurons using brian simulator.

[34] Golomb D. (2007). Neuronal synchrony measures. *Scholarpedia*.

